# Acute internal medicine in focus: The people, the purpose, the progress

**DOI:** 10.1016/j.fhj.2026.100533

**Published:** 2026-06-25

**Authors:** Vicky Anne Price

**Affiliations:** Liverpool University Hospital Trust, United Kingdom

**Keywords:** Acute internal medicine, Acute medical unit, Same-day emergency care, Urgent and emergency care, Patient flow

## Abstract

Acute internal medicine (AIM) emerged in the 1990s in response to rising emergency admissions, constrained hospital capacity and fragmented models of care. This article traces the development of the specialty from its early conceptualisation through to its current role within modern urgent and emergency care systems. It explores the evolution of acute medical units, the expansion of ambulatory and same-day emergency care, the introduction of enhanced care areas and the maturation of a distinctive training pathway. Drawing on personal experience and national data, the article highlights how AIM has developed a unique skillset focused on managing undifferentiated illness, maintaining patient flow, and delivering patient-centred care in high-pressure environments. In the context of increasing multimorbidity and system strain, AIM is positioned not as an optional organisational model, but as a core clinical and operational mechanism essential to safe, efficient and sustainable acute care delivery.

The widespread portrayal of NHS hospitals as being ʻin crisis’ has become a familiar headline in recent years. For many clinicians, this sense of pressure feels familiar. Similar narratives dominated the 1990s, describing an urgent and emergency care system struggling with capacity, rising demand and repeated structural reform. As Jean-Baptiste Alphonse Karr observed in 1849, ʻ*Plus ça change, plus c’est la même chose.*’

It was during the 1990s that the idea of acute medicine first emerged. The concept was not overly complex: to cohort all new medical admissions in one place, cared for by a dedicated team for the first 72 h of their hospital stay. Crucially, this was always envisaged as a multidisciplinary endeavour, with dedicated nurses, physiotherapists, pharmacists and occupational therapists working alongside doctors. Together, they would ensure that patients received the best possible care during those first critical hours. As enthusiasm grew, so did the recognition of the importance of a bespoke training programme to ensure that people had the requisite skillset to work in these areas.

The concept of acute medicine was dismissed by many as a ʻnon-specialty’ – or just another iteration of general medicine. Yet evidence soon emerged showing that hospitals adopting acute medical units (AMUs) saw reductions in length of stay, lower mortality and improved patient satisfaction.^1^, ^2^

Questions have also arisen about the need for both emergency medicine and acute internal medicine. These are now well-established as distinct but complementary specialties working together across urgent and emergency care. Delivering safe, efficient acute care requires expertise across the emergency–acute pathway, supported by both the Royal College of Emergency Medicine and the Society for Acute Medicine. Emergency medicine provides rapid assessment and stabilisation at the hospital front door, while acute internal medicine (AIM) leads the early inpatient phase of care, including diagnostic clarification, management of complexity, and expert decision-making over time. These roles are interdependent: effective patient flow relies on strong emergency care upstream and well-functioning AMUs and same-day emergency care downstream. Collaboration between specialties remains essential to improve outcomes for acutely unwell adults and I have been fortunate to witness the results of positive collaboration on both local and national levels.

I was part of the first cohort of acute medicine trainees in my region. Despite some enthusiasm for the model, scepticism was common. I recall being told, while travelling to my specialty registrar interview, that choosing acute medicine was a mistake and that the specialty would not endure. Instead, I have been proud to witness its steady growth: the development of ambulatory care, the introduction of enhanced care areas, and the expansion of a distinctive skillset that now includes point-of-care ultrasound (POCUS). Importantly, I have seen the impact that effective acute care can have not only on individual patients, but on whole hospital systems.

## From birth to 25 years – the progress

In 1989–90, approximately 270,000 hospital beds were available in NHS hospitals in England, but by 1999 this number had fallen to around 190,000.^3^ At the same time, emergency admissions were rising by approximately 2% per year.^4^, ^5^, ^6^, ^7^ These diverging trends placed growing strain on hospital capacity and exposed inefficiencies in traditional models of care.

Patients arrived with a wide range of medical conditions and were inevitably scattered across whichever wards had capacity (illustrated later in Box 2). The post-take ʻsafari ward round’ was a familiar sight: consultants striding at impossible speed through hospital corridors, junior doctors trailing behind with clipboards, multiple request forms and mounting anxiety as they tried to keep up.

**Fig. 1:**
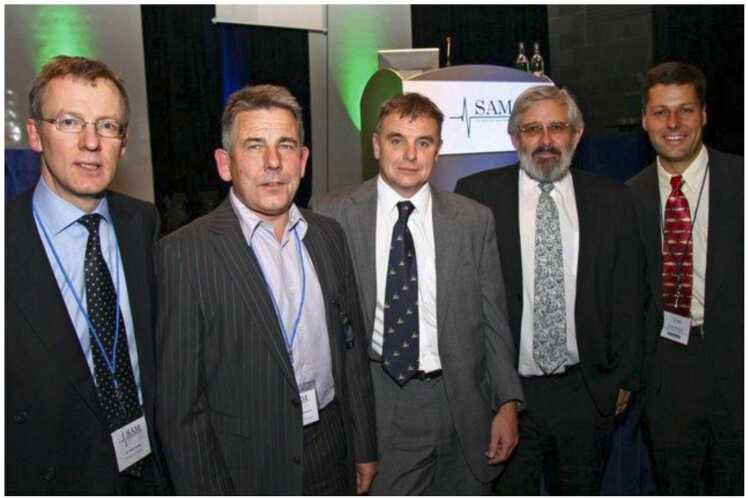
The five clinicians who founded the Society for Acute Medicine (from left to right: Mike Jones, Paul Jenkins, Derek Bell, Rhid Dowdle and Chris Roseveare).

The inefficiency of this system was obvious. A small group of forward-thinking clinicians began to articulate a different model: a unit where all acute medical admissions could be cared for in one place, under the leadership of a ʻconsultant of the day’. From this, the acute medical unit (AMU) was born (Fig. 1).

Reproduced with kind permission of Rhid Dowdle. Image originally published on the Society for Acute Medicine website.^8^

Rhid Dowdle has eloquently described the struggles of these early years in his writings on the history of the Society for Acute Medicine, available on the Society for Acute Medicine’s website.^8^

Despite initial resistance to recognising AIM as a specialty, the number of AMUs grew, alongside the evidence base supporting them.^1^, ^2^ In 2000, the Society for Acute Medicine (SAM) was formally launched, rapidly becoming a strategic body representing the multidisciplinary workforce committed to advancing the specialty.

Recognition of the specialty and its skillset (Box 1) soon followed.

Box 1 **Definition of acute medicine.**

**Table:** 

Acute internal medicine is a specialty that operates within the acute medical unit and same-day emergency care unit at the front door of the hospital where the multidisciplinary team works together to provide: • Early senior clinical decision-making for undifferentiated, often complex presentations • Management of acute illness in patients with multimorbidity and frailty • Stabilisation, investigation and initiation of treatment • Clear planning: discharge (including same-day discharge), transfer to other specialty team, or escalation to enhanced or critical care • Person-centred care, including discussions about goals of care and ceilings of treatment Acute physicians optimise care early, reduce delays, and ensure that patients are directed to the right care pathway, in the right setting, first time.

In April 2004, *Acute medicine: making it work for patients*, a report by a Royal College of Physicians (RCP) working party, was published, signalling formal endorsement of the specialty after earlier hesitation.^9^

## The development of the people and the purpose

### Development of ambulatory care (later known as same-day emergency care, SDEC)

Diagnostic acumen has always been central to acute medicine, but another defining skill quickly became apparent: pragmatism, grounded in patient-centred care. For some patients, prolonged hospital admission was not only undesirable but actively harmful.

In the early 2000s, I was admitting patients with conditions such as deep vein thrombosis for 7 days. The ambulatory care model (later renamed SDEC) challenged this practice and demonstrated that patients with a range of conditions could be managed safely at home. The launch of the *Directory of ambulatory emergency care* in 2007 identified several conditions that could be managed in this way.^10^ This shift proved beneficial for patients and helped relieve pressure on inpatient beds, and acute medical teams were ideally placed to lead this model of care.^11^ SAM’s Benchmarking Audit (SAMBA) in 2023 showed that in 35.5% of units, a third or more of acute medical admissions were managed through SDEC.^12^

### Development of enhanced care

One of the skillsets of acute physicians is to care for critically unwell patients. Training therefore includes time in intensive care to develop skills in managing patients at the most severe end of illness. As early as 2007, the RCP report *Acute medical care – the right person, in the right setting, first time* recommended the introduction of enhanced care areas, distinct from critical care, for patients requiring higher-intensity monitoring.^13^

Since then, increasing numbers of AMUs have developed enhanced care areas, facilitating timely transfer from pressured areas such as emergency department resuscitation rooms. In SAMBA 2023, 24% of participating units had an enhanced care area.^9^

This progress has been underpinned by joint guidance with the Intensive Care Society (ICS),^14^ alongside recognition of the essential role of the whole multidisciplinary team, including strong endorsement from the Royal College of Nursing in defining and developing the required nursing skillset.

### Development of training

As the specialty evolved, the need for a structured training pathway became clear. A higher medical training curriculum for sub-specialty training in acute medicine was published in 2003 by the Joint Committee on Higher Medical Training. This was followed by the introduction of the general internal medicine (AIM) curriculum in 2007, and formal recognition of AIM as a distinct specialty by the General Medical Council in 2009, with a dedicated curriculum published the same year. The most recent curriculum update, implemented in 2022, incorporated point-of-care ultrasound (POCUS).

The competencies required to deliver high-quality acute care are distinctive. They include managing uncertainty and risk, maintaining patient flow, leading high-turnover environments and advanced clinical reasoning. These are developed within the AIM training programme and are unique to the specialty.

We have published guidance on the future appointment of clinicians working in acute medicine, emphasising the importance of recognising and valuing this unique skillset and recruiting appropriately trained physicians into these roles.

### The purpose of acute medicine in 2025

These advances in standards of training, and increased recognition, are timely. The NHS faces unprecedented pressure from an ageing population and increasing multimorbidity.^15^ The complications of both disease and treatment frequently present as acute medical emergencies, placing significant demands on urgent and emergency care services. This requires highly trained acute physicians who can assess complex patients rapidly, deliver effective treatment, and engage in honest and compassionate conversations about goals of care.

Expertise in managing undifferentiated acute illness has expanded alongside multidisciplinary innovation and collaboration, enabling acute medicine to develop at pace. In 2025, SAM published updated guidance defining the role of the specialty.^16^

As I noted above, acute medicine has had to fight the persistent misconception is that it is the same as general medicine. SAM has worked hard to clarify that acute medicine is a specialty and the acute take as a service are not the same and should not be conflated. Dedicated guidance outlines these differences.^17^

When AMUs are used appropriately, the benefits for patient outcomes, flow and system efficiency are as clear now as they were in the early 2000s.^18^ From the days of the safari ward round, acute medicine has moved us forward to enable the delivery of patient-centred care in the most suitable areas (Box 2, Box 3). All patient names and details in Boxes 2, 3 and 4 are fictional and used for illustrative purposes only.

Box 2 **Example ʻsafari’ patient list PTWR 1990.**

**Table:** 

**Ward 1** Doris (67) Pneumonia Mohammed (65) New atrial fibrillation **Ward 7B** John (43) Cellulitis of left leg **Ward 3** Fatima (21) Diabetic ketoacidosis **Ward 9** Graham (83) Urosepsis (not for ITU) Peter (46) Alcohol withdrawal **Ward 12** Mary (72) Fall with long lie Maryam (35) Paracetamol overdose **Ward 23C** Amit (40) Chest pain Jacob (70) ?Pulmonary embolism **Ward 4RD** Jane (23) Headache **Ward 8 (south)** Sofia (56) Anaemia Michael (53) ?Guillain–Barre **Ward A6** Finbar (68) Jaundice Zainab (70) Renal failure

Box 3 **Example patient list 2025.**

**Table:** 

**SDEC → home** Mohammed (65) New atrial fibrillation John (43) Cellulitis of left leg Amit (40) Chest pain Jacob (70) ?Pulmonary embolism Jane (23) Headache Sofia (56) Anaemia **Enhanced care** Fatima (21) DKA Graham (83) Urosepsis Michael (53) ?Guillain–Barre **AMU** Peter (46) Alcohol withdrawal Maryam (35) Paracetamol overdose Doris (67) Pneumonia Finbar (68) Jaundice Zainab (70) Acute kidney injury **Acute frailty** Mary (72) Fall with long lie

## Summary

Acute medicine is one of the newer medical specialties; however, it is now firmly established with a clearly defined remit and a growing evidence base. National guidance, including the 2024 GIRFT report, reinforces the principles underpinning high-quality acute medical care.^19^

The AIM training programme delivers bespoke education designed to develop highly skilled acute physicians, capable of managing a diverse patient population efficiently while delivering patient-centred care.

Acute medicine is not an optional organisational preference, it is the clinical and operational mechanism that makes modern acute care safe, effective and financially sustainable in a system facing rising demand, complexity and constrained resources.

When AMUs are bypassed or compromised by operational pressures, such as when the acute medical team is transplanted into the emergency department, patients lose access to early specialist care, avoidable admissions increase and flow deteriorates. Staff morale and patient outcomes suffer. When used as intended, AMUs improve patient experience, support safe discharge (Box 4), enhance system efficiency and provide a vital educational environment.

Box 4 **A tale of two pathways – how system design shapes patient experience.**

**Table:** 

Sofia, a 56-year-old woman, presented to her general practitioner with several weeks of fatigue. Routine blood tests, reported at 19:00 on the Friday of a bank holiday weekend, revealed a haemoglobin concentration of 46 g/L and markedly deranged liver function tests. **Pathway 1: admission via the emergency department** Sofia was referred urgently to the emergency department. She was haemodynamically stable on arrival but, due to crowding and limited inpatient capacity, waited over 12 h in the department before a bed became available. She was admitted for blood transfusion and investigation, including cross-sectional imaging. During a prolonged inpatient stay, she was diagnosed with metastatic bowel cancer. On day 14 she developed hospital-acquired pneumonia, extending her admission by a further week and delaying the initiation of cancer treatment. Prolonged hospitalisation, uncertainty and loss of autonomy contributed to a deterioration in her mood and increasing feelings of isolation from her family. **Pathway 2: coordinated acute medical care via** s**ame**-d**ay** e**mergency** c**are** In an alternative pathway, Sofia’s general practitioner contacted the on-call acute physician directly, and next-day review was arranged in the same-day emergency care (SDEC) unit. She received three units of blood and underwent focused point-of-care ultrasound, which demonstrated liver metastases. The findings were discussed sensitively with Sofia and her family, and she returned home later that day with their support. A CT scan arranged for the following Monday confirmed likely bowel cancer, and she was promptly referred to the colorectal multidisciplinary team, allowing further diagnostic tests, oncology review, and initiation of systemic treatment without unnecessary delay. **Reflection** Both pathways led to the same diagnosis and oncological referral. However, for Sofia, the second approach avoided prolonged hospitalisation at a critical point in her illness, reduced exposure to hospital-acquired harm, and preserved dignity, autonomy and family support. This case illustrates how acute medicine, embedded within integrated urgent care pathways, can transform not only clinical outcomes but patient and family experience, while supporting wider system flow

Optimising AMUs and adhering to the acute medicine model of care offers us a critical opportunity to meet current and future challenges. With talented physicians coming through training and the passion evident in units across all four nations, I have no doubt that acute medicine will continue to adapt, thrive and deliver excellent patient care for years to come.

## CRediT authorship contribution statement

**Vicky Anne Price:** Conceptualization, Writing – original draft, Writing – review & editing.

## Funding

This research did not receive any specific grant from funding agencies in the public, commercial or not-for-profit sectors.

## Declaration of competing interest

Dr Vicky Price is the current president of the Society for Acute Medicine.
